# Non-invasive assessment of muscle spasticity in children with cerebral palsy undergoing botulinum toxin treatment using near-infrared spectroscopy

**DOI:** 10.1186/s12984-025-01853-z

**Published:** 2025-12-22

**Authors:** Mehdi Nourizadeh, Maria Juricic, Jocelyn Begin, Leila Bektash, Stacey Miller, Kishore Mulpuri, Babak Shadgan

**Affiliations:** 1 International Collaboration on Repair Discoveries, Vancouver, Canada; 2 Department of Orthopaedics, University of British Columbia, Vancouver, Canada; 3 Department of Orthopaedic Surgery, BC Children’s Hospital, University of British, Columbia, Canada; 4 Department of Pathology & Laboratory Medicine, University of British Columbia, Vancouver, Canada

**Keywords:** Cerebral palsy, Muscle spasticity, Botulinum toxin (BoNT-A), Near-Infrared spectroscopy (NIRS), Tissue oxygenation index (TOI%), Spasticity assessment

## Abstract

**Background:**

Spasticity is a common and debilitating feature of cerebral palsy (CP), often treated with botulinum toxin A (BoNT-A). Standard assessments, such as the Modified Ashworth Scale (MAS), are subjective and prone to inter-rater variability. Near-infrared spectroscopy (NIRS) is a non-invasive method that measures muscle oxygenation and hemodynamics. In CP, spastic muscles exhibit impaired perfusion and abnormal oxygen consumption due to sustained contraction and elevated intramuscular pressure. Prior studies suggest that reducing spastic overactivity improves local tissue oxygenation, indicating that NIRS-derived metrics could objectively reflect spasticity severity and treatment response.

**Objective:**

To evaluate the feasibility of using NIRS as a biomarker for spasticity severity and treatment response in children with CP receiving BoNT-A and to assess the correlation between NIRS-derived tissue oxygenation index (TOI%) and MAS scores over time.

**Methods:**

In this prospective pilot study, eight children (aged 2–17 years) with CP undergoing BoNT-A treatment were assessed. Muscle oxygenation was recorded using a portable NIRS device at baseline, 2–4 weeks, 6–10 weeks, 3 months, and 6 months post-injection. Spasticity was measured concurrently using MAS. Temporal changes in TOI% and MAS were analyzed using repeated-measures ANOVA, and associations between the two were evaluated with Pearson correlation.

**Results:**

TOI% increased significantly after injection, peaking at 6–10 weeks and 3 months (*p* < 0.001), then declined toward baseline by 6 months. MAS scores showed a similar trend, with reduced spasticity during peak effect. A moderate, significant negative correlation between TOI% and MAS (*r* = − 0.409, *p* < 0.05) indicated higher oxygenation was associated with lower spasticity.

**Conclusion:**

NIRS is a feasible, non-invasive tool for assessing muscle spasticity and monitoring BoNT-A response in children with CP. Its correlation with MAS supports physiological relevance and suggests NIRS may offer a more objective measure of spasticity. Larger studies are warranted to validate these findings and support clinical use.

## 1. Introduction

Cerebral palsy (CP) is the most prevalent motor disability in childhood, affecting approximately 2–3 per 1,000 live births worldwide [1–3]. Spasticity, a hallmark of CP, is characterized by a velocity-dependent increase in muscle tone due to hyperexcitable stretch reflexes, resulting from upper motor neuron dysfunction [1–3]. Clinically, it manifests as involuntary muscle stiffness, hyperreflexia, and impaired motor control, significantly limiting mobility, daily functioning, and overall quality of life [1–3]. If left unmanaged, spasticity can lead to muscle contractures, joint deformities, and chronic pain, further exacerbating disability and dependency [3].

Effective spasticity management is essential to optimize motor function and promote participation in daily activities [4]. Current treatment options include physical and occupational therapy, oral antispasmodic medications, intrathecal baclofen administration, and surgical interventions. Among these, intramuscular injections of botulinum toxin A (BoNT-A) remain a widely used first-line treatment due to their ability to temporarily reduce muscle overactivity and improve functional mobility [4–8]. However, optimizing therapeutic outcomes requires a precise assessment of spasticity severity to determine the appropriate dosage, time of injection and injection sites [8]. Inaccurate or inconsistent evaluation may lead to suboptimal treatment outcomes, reinforcing the need for reliable and objective spasticity assessment tools [9, 10].

Spasticity evaluation commonly relies on clinical rating scales such as the Modified Ashworth Scale (MAS) and the Tardieu Scale [9]. [10] Although widely used, these tools are inherently subjective and susceptible to inter-rater variability. Objective techniques such as electromyography (EMG), elastography, and biomechanical assessments have been explored, but each presents limitations in accessibility, complexity, or specificity [9, 10]. A recent review highlights the lack of a universally accepted, standardized, and easily applicable tool for assessing spasticity in CP, underscoring the critical need for objective, non-invasive, and reliable methods [9, 10].

Near-infrared spectroscopy (NIRS) has emerged as a promising optical modality for evaluating muscle physiology and function [11, 12]. By quantifying relative concentrations of oxygenated and deoxygenated hemoglobin in muscle tissue, NIRS can measure and monitor changes in regional tissue oxygenation and hemodynamics [11, 12]. In a spatially-resolved configuration, NIRS can measure the absolute level of tissue oxygenation in percentage (TOI%) [13].

Spasticity is not only a disorder of motor control but also produces characteristic changes in the local muscle microenvironment. Voluntary isometric muscle contraction [23] and sustained involuntary contraction elevate intramuscular pressure and mechanically compress the capillary bed, thereby restricting local blood flow and oxygen delivery to muscle fibres [28]. Experimental and human studies demonstrate that changes in contraction force and motor activation are closely accompanied by changes in muscle oxygenation measured with NIRS, supporting the physiological sensitivity of NIRS to altered muscle perfusion and metabolic demand [11, 21]. Moreover, interventions that reduce tonic muscle overactivity, such as botulinum toxin injections, can restore aspects of muscle perfusion and oxygen availability, providing further rationale for using NIRS to monitor treatment effects [22, 27]. Although the underlying mechanism appears similar in children and adults, developmental differences in muscle architecture and vascular regulation may affect the magnitude and variability of the hemodynamic response in pediatric cerebral palsy; accordingly, direct pediatric validation is required [24]. 

NIRS has gained widespread use in both clinical and research settings due to its non-invasive nature, portability, and capacity for continuous, real-time monitoring of tissue oxygenation and hemodynamics [14]. In medicine, NIRS is commonly applied to monitor cerebral oxygenation in neonatal [15] and adult intensive care units [16], assess tissue viability during reconstructive surgeries [17], evaluate regional perfusion in patients with peripheral vascular disease [5], study muscle injuries [18], diagnose bladder dysfunction [19], and monitor spinal cord injury [20]. In exercise science, NIRS is frequently used to study skeletal muscle oxygenation dynamics during physical activity, evaluate muscle fatigue, and determine the anaerobic threshold during incremental exercise [21, 22]. Its ability to measure localized muscle metabolism under various physiological conditions makes NIRS particularly valuable in sports performance monitoring, rehabilitation, and neuromuscular research. Given that spastic muscles exhibit altered blood flow and oxygen consumption patterns, NIRS may serve as an indirect yet sensitive tool to evaluate spasticity severity and treatment response [23]. 

This pilot study aimed to investigate the feasibility of using NIRS to assess and monitor muscle spasticity in children with CP undergoing BoNT-A treatment. Specifically, we aim to quantify longitudinal changes in muscle tissue oxygenation following injection and to evaluate correlations with MAS scores. Based on the known hemodynamic consequences of spasticity, namely reduced muscle perfusion and impaired oxygen utilization due to sustained contraction and elevated intramuscular pressure [12, 19, 26], we hypothesized that reducing spasticity with botulinum toxin A would improve local muscle oxygenation, as measured by NIRS. We expected that increases in NIRS-derived TOI% following treatment would be associated with reductions in spasticity as measured by the MAS. Our findings may support the development of a more objective, physiologically grounded approach to assessing and monitoring spasticity.

## Methods

### Study design

This prospective pilot observational study evaluated changes in muscle oxygenation and spasticity severity in children with CP receiving BoNT-A treatment. Baseline measurements were obtained before injection, followed by serial post-injection assessments at predefined time points. The study was approved by the Clinical Research Ethics Board of the University of British Columbia, and written informed consent was obtained from parents or legal guardians. All participant data were de-identified and securely stored to ensure confidentiality.

### Participants

Eight children diagnosed with CP (aged 2–17 years) were enrolled. Inclusion criteria included: (1) presence of spasticity in at least one limb; (2) no prior surgical intervention on the target muscle; and (3) no contraindication to NIRS monitoring or BoNT-A injection. Informed consent was obtained from all parents or legal guardians prior to enrollment.

### Instrumentation

Muscle tissue oxygenation was measured using a spatially resolved NIRS device (PortaLite, Artinis Medical Systems, The Netherlands). The NIRS probe was positioned on the belly of the target spastic muscle receiving BoNT-A injection (e.g., gastrocnemius, hamstrings, rectus femoris, adductors). To ensure consistency, the probe location was marked during the baseline assessment and replicated in subsequent sessions. The probe was secured with elastic straps and covered with an opaque shield to minimize motion artifacts and ambient light interference.

### Study protocol

Although target muscles differed among participants according to clinical indication, each child was measured repeatedly on the same muscle across all study time points. This within-subject longitudinal approach minimized inter-muscle variability in oxygenation measurements.

#### Baseline assessment

Prior to BoNT-A injection, baseline muscle oxygenation (Time 1) was recorded using NIRS while participants were at rest. All resting-state recordings were performed with participants in a relaxed supine position, with the targeted limb supported to minimize involuntary movement and muscle activation. NIRS signals were recorded continuously for three minutes, and spasticity was concurrently assessed using the MAS.

#### Potulinum toxin injection

BoNT-A (Dysport^®^, Ipsen Biopharm Ltd., UK) was administered by a pediatric orthopedic specialist according to established clinical guidelines. Dosage and injection sites were determined individually based on standard clinical assessment and best practices for spasticity management.

#### Follow-up assessments

Follow-up assessments were performed at four post-injection time points: 2–4 weeks (Time point 2), 6–10 weeks (Time point 3), 3 months (Time point 4), and 6 months (Time point 5). At each session, muscle oxygenation was measured for three minutes under resting conditions by NIRS, and spasticity was reassessed using the MAS.

### NIRS data processing

Tissue oxygenation index (TOI%) was calculated automatically by the NIRS device using spatially resolved spectroscopy according to the formula:

$${\text{TOI}}{\%}=\frac{{\text{O}}2{\text{Hb}}}{{\text{O}}2{\text{Hb}}+{\text{HHb}}} \times100$$

Data acquisition and visualization were performed using OxySoft software (Artinis Medical Systems, The Netherlands). No additional signal preprocessing was applied. For each measurement, the TOI% value used for analysis corresponded to the first stable reading obtained immediately after sensor placement and signal stabilization. This approach minimized motion artifacts during longer recordings and ensured that measurements reflected a relaxed resting state.

### Statistical analysis

Temporal changes in TOI% over time across gender and Dysport dosage were analyzed using a factorial repeated measures analysis of variance (RM-ANOVA). The normality of residuals was tested using the Shapiro–Wilk test (*p* > 0.05), and sphericity was evaluated via Mauchly’s test (*p* > 0.05). MAS scores were analyzed using a one-way repeated-measures ANOVA. Post hoc pairwise comparisons were Bonferroni-adjusted. Post hoc comparisons were adjusted using Bonferroni, allowing to compare TOI% across time points where meaningful and significant changes in TOI% occur. Pearson correlation analysis was used to evaluate the direction and strength of the relationship between TOI% and MAS scores. Data analyses were performed using R software, with significance set at *p* < 0.05.

## Results

### Participant characteristics

Eight children with CP (age range: 2–17 years; 3 females, 5 males) completed the study. Table 1 summarizes participant demographics, including age, gender, weight, Gross Motor Function Classification System (GMFCS) level, target muscle and Dysport dosage. Participants received either 150 or 300 units of Dysport, with target muscles including the gastrocnemius, hamstrings, rectus femoris, and adductor group.

**Table 1:** The distribution of participants based on GMFCS level and dysport dosage

Participants	Age	Gender	Weight (Kg)	GMFCS Level	Dysport Dosage	Target Muscle
P01	5Y 9 M	F	13.8	5	150 Units	Left Adductor
P02	8Y 8 M	M	24.4	4	150 Units	Right Hamstring
P03	16Y 11 M	M	80.6	4	300 Units	Right Rectus Femoris
P04	17Y 10 M	F	48.7	4	300 Units	Right Gastrocnemius
P05	2Y 11 M	M	13.9	1	150 Units	Right Gastrocnemius
P06	2Y 11 M	M	13.9	1	150 Units	Left Gastrocnemius
P07	6Y	M	19.3	2	300 Units	Right Gastrocnemius
P08	7Y	F	39.4	2	300 Units	Left Gastrocnemius

### Muscle oxygenation changes over time

Muscle oxygenation, measured as the TOI% using NIRS, showed significant temporal variation during the six-month follow-up. Table 2 presents descriptive statistics and the results of the normality test. TOI% values increased after BoNT-A injection, peaking at 6–10 weeks (Time Point 3) and at 3 months (Time Point 4), before returning toward baseline by 6 months (Time Point 5). Shapiro–Wilk tests confirmed normal distribution of TOI% values (*p* > 0.05 for all time points).

**Table 2:** Descriptive statistics and normality test for TOI% measurements at five time Points. Over 6 months Post-Injection

Time Point	Minimum TOI	Maximum TOI	Mean TOI	Std. Deviation	Shapiro-Wilk test (*p* > 0.05)
Baseline (Time-Point 1)	40	63	54.2	7.99	0.50
2–4 weeks (Time-Point 2)	51	66	61.2	5.54	0.13
6–10 weeks (Time-Point 3)	50	70	63.4	6.85	0.10
3 months (Time-Point 4)	50	69	62.1	5.74	0.18
6 months (Time-Point 5)	44	61	55.3	5.91	0.13

**Fig. 1:**
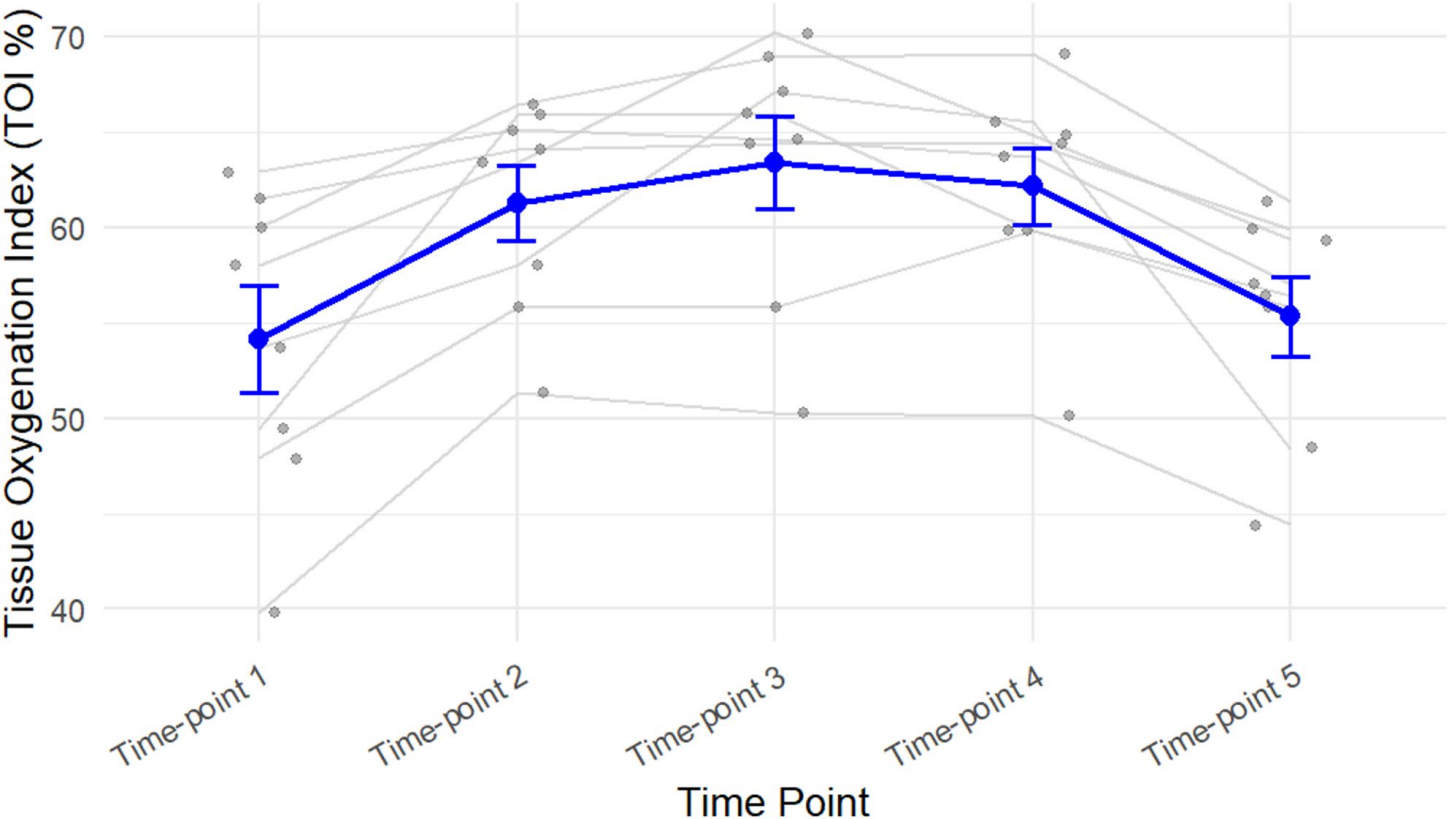
The trend of TOI% values per participant over time, highlighting the peak improvements and subsequent decline. Thin gray lines represent individual participants’ TOI% trajectories over time. Gray dots indicate individual participant values. Blue dots show the mean TOI% at each time point, with blue error bars representing the standard error of the mean. The blue line connects the mean TOI% values across time points, illustrating the overall trajectory

Figure 1 illustrates the estimated marginal means of TOI% across five time points. There is an initial increase within the first 2 to 4 weeks (Time point 2), the mean increases from 54.2 to approximately 61.2, indicating an improvement in muscle oxygenation changes early in the observation period. By the third session (6 to 10 weeks), the mean muscle oxygenation further increases to around 63.4, indicating that the highest level of oxygenation change is observed during this period. However, at the fourth (three months) and fifth sessions (six months), the mean returned to baseline, 62.1 and 55.3, respectively.

#### Statistical analysis of muscle oxygenation driven by NIRS sensors

A factorial repeated-measures ANOVA revealed a significant main effect of time on muscle oxygenation (F (4,16) = 12.552, *p* < 0.001, η² = 0.132). However, neither gender F (1,4) = 0.483, *p* > 0.05, η² = 0.029) nor Dysport dosage (F (1,4) = 0.188, *p* > 0.05, η² =0 0.011) had significant main effects on muscle oxygenation changes. No significant interaction effects for muscle oxygen changes have been observed across gender over time (F (4,16) = 0.813, *p* > 0.05, η² = 0.009), across Dysport dosage over time (F (4,16) = 0.643, *p* > 0.05, η² = 0.007), and across Dysport dosage and gender categories over time (F (4,16) = 0.561, *p* > 0.05, η² = 0.006). Thus, the pattern of muscle oxygenation changes over time did not differ across Dysport dosages, gender categories, or their interactions.

**Table 3:** Factorial Repeated-Measures ANOVA for changes in TOI% over six months

Effects	Sum of Squares	df	Mean Square	F	*p*	effect size(η²)
Within Subjects						
Time	525.5	4	131.38	12.552	< 0.001	0.132
Time ✻ Dysport	26.9	4	6.73	0.643	0.640	0.007
Time ✻ Gender	34.1	4	8.51	0.813	0.535	0.009
Time ✻ Dysport ✻ Gender	23.5	4	5.87	0.561	0.694	0.006
Residual	167.5	16	10.47			
Between Subjects						
Dysport	45.7	1	45.7	0.188	0.687	0.011
Gender	117.3	1	117.3	0.483	0.525	0.029
Dysport ✻ Gender	129.4	1	129.4	0.533	0.506	0.032
Residual	971.0	4	242.7			

Post hoc pairwise comparisons with Bonferroni correction (Table 4) revealed that TOI% increased significantly from baseline (Time point 1) to 2–4 weeks (Time point 2) (*Mean Difference = -7.21*, *p* 0 < 0.01), 6–10 weeks (Time point 3) (*Mean Difference = -9.73*, *p* < 0.001), and three-month (Time point 4) (*Mean Difference = -8.55*, *p* < 0.01), confirming a substantial rise in muscle oxygenation was observed between baseline and three-month follow-up However, muscle TOI% significantly decreased from Time point 3 to Time point 5 (*Mean Difference = 8.27*, *p* < 0.01). Similarly, Muscle TOI% showed a significant drop from Time point 4 to Time point 5 (*Mean Difference = 7.09*, *p* < 0.01).

**Table 4:** Pairwise comparisons of muscle oxygenation changes over time

Time	Time	Mean Difference	SE	df	t-statistic	*p*_bonferroni_
Time-Point 1	**Time-Point 2**	-7.21	1.75	16	-4.128	0.008
	**Time-Point 3**	-9.73	1.75	16	-5.570	< 0.001
	**Time-Point 4**	-8.55	1.75	16	-4.894	0.002
	Time**-**Point 5	-1.46	1.75	16	-0.837	1.000
Time-Point 2	Time**-**Point 3	-2.52	1.75	16	-1.442	1.000
	Time**-**Point 4	-1.34	1.75	16	-0.766	1.000
	**Time-Point 5**	5.75	1.75	16	3.291	0.046
Time-Point 3	Time**-**Point 4	1.18	1.75	16	0.676	1.000
	**Time-Point 5**	8.27	1.75	16	4.733	0.002
Time-Point 4	**Time-Point 5**	7.09	1.75	16	4.057	0.009

**Fig. 2:**
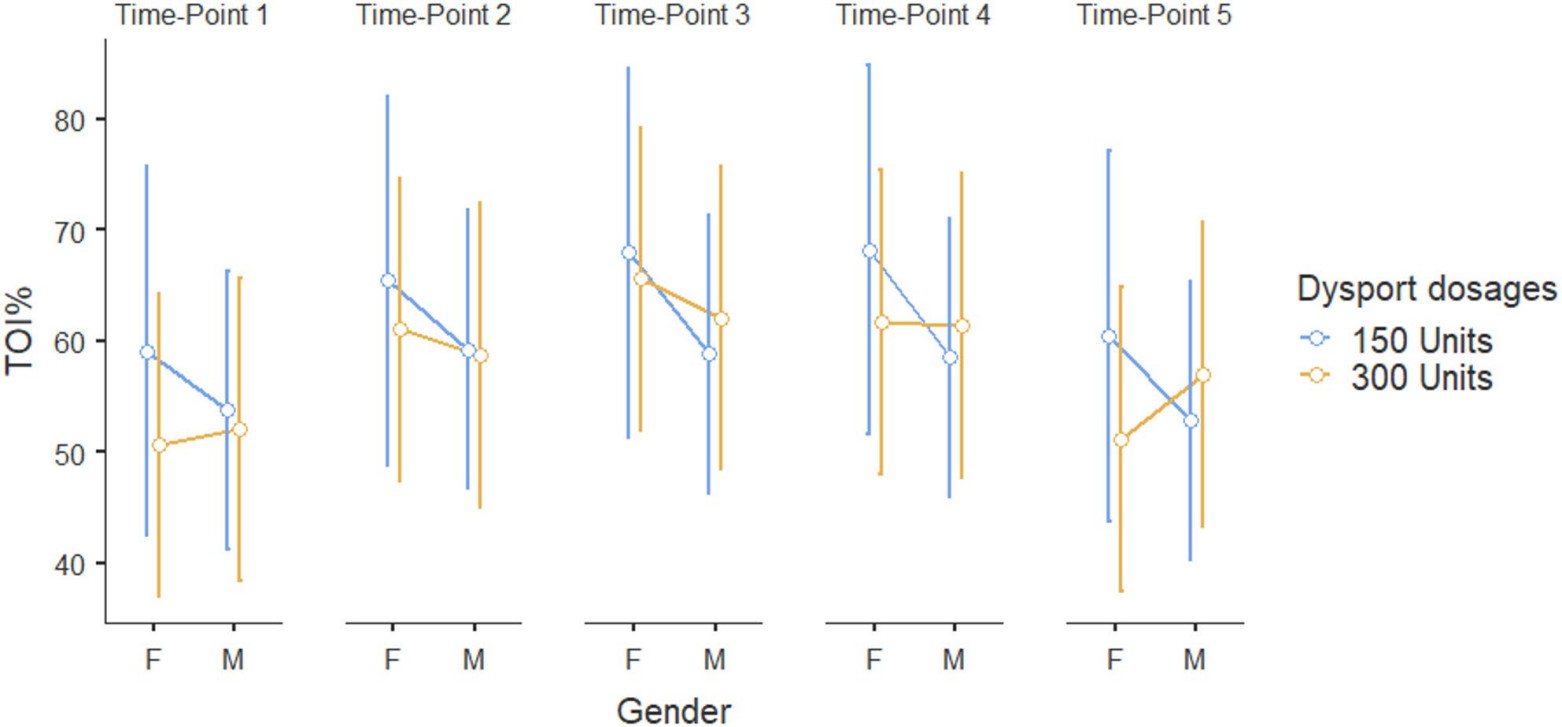
The estimated marginal means of TOI% across time for different Dysport dosage and gender groups. Blue lines represent TOI% values for a Dysport dosage of 150 units. In comparison, orange lines represent TOI% values for 300 units across males and females, with error bars representing the standard error of the mean

**Fig. 3:**
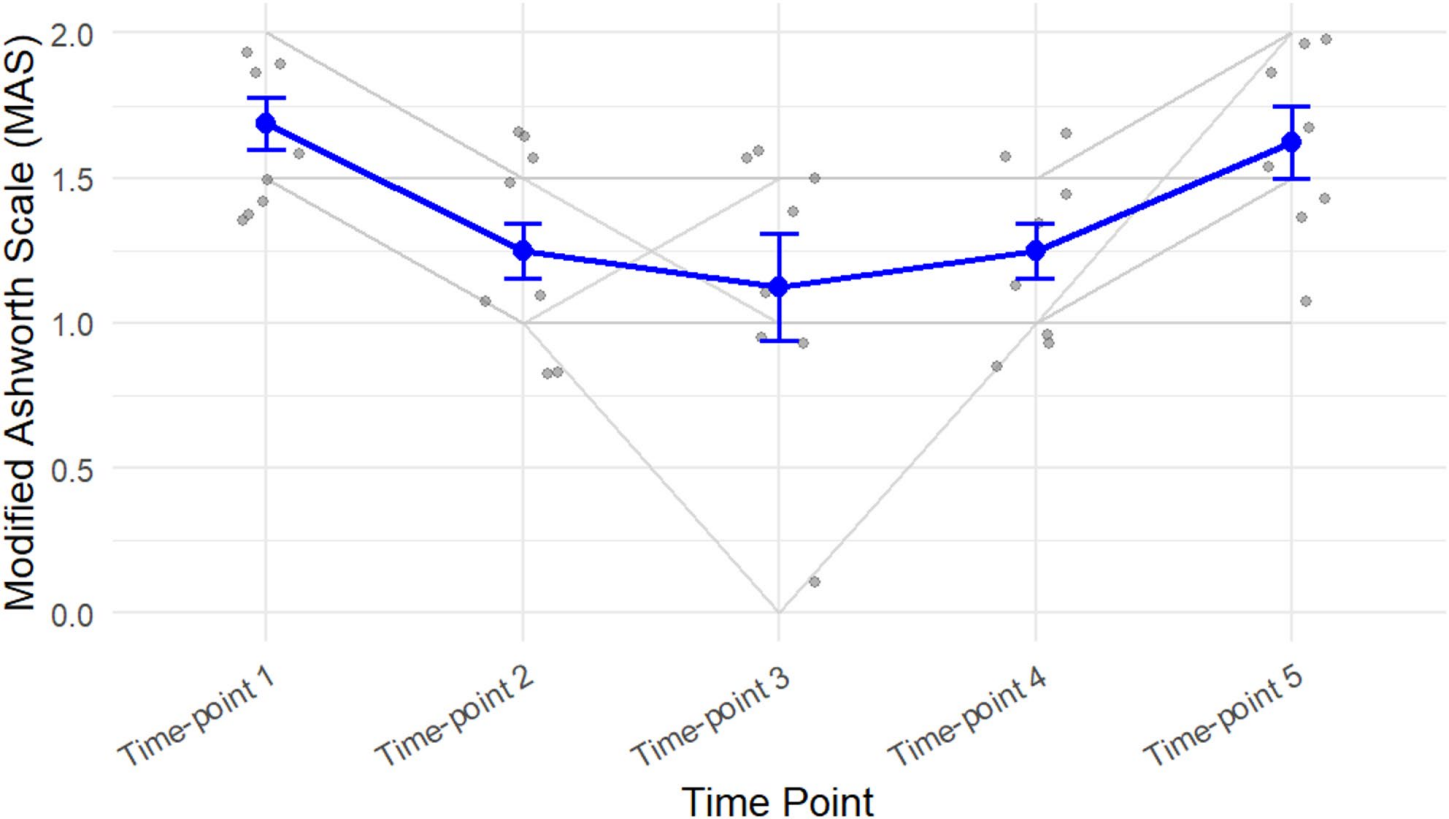
Depicts MAS trends over the study period, showing meaningful reductions at key intervals. The thin gray lines represent the MAS scores for each participant over time. Gray dots indicate individual participant values. Blue dots show the mean MAS at each time point, with blue error bars representing the standard error of the mean. The blue line connects the mean MAS values across time points, illustrating the overall trajectory

According to Fig. 2, it is evident that muscle oxygenation peaked around Time point 3–4 and declined at Time point 5, suggesting a short-term improvement followed by a decrease by Time point 5. Even though, the Repeated Measures ANOVA analysis (Table 3) did not result in a significant interaction affect of gender and Dysport dosage in TOI% values over time, Fig. 3 suggests potential interaction effects gender and Dysport dosage in TOI% values; however, due to the small sample size, these findings are not conclusive. A larger sample in future studies will be necessary to validate these observations and determine their statistical significance.

### 3.3 MAS changes over time

One-Way Repeated Measures ANOVA for Spasticity levels across five time points revealed that Spasticity levels, as measured by the Modified Ashworth Scale (MAS), significantly changed over time (F (4,28) = 7.75, *p* < 0.001, η² = 0.363), indicating a moderate to large effect size (Table 5).

**Table 5:** Results of the one-way repeated measures ANOVA: spasticity levels measured by MAS over six months

Within-Subjects Effects	Sum of Squares	df	Mean Square	F	*p*	effect size(η²)
Time-Point	2.31	4	0.578	7.75	< 0.001	0.363
Residual	2.09	28	0.075			

Post hoc analysis showed significant differences in MAS levels between time-point 1 and time-point 2, 3, and 4 (*p* < 0.05). Besides, the third session and fifth session statistically differ in Spasticity levels (*p* < 0 0.01). The other comparisons did not reach meaningful differences (Table 6).

**Table 6:** Post hoc pairwise comparisons of spasticity levels measured by MAS over six months

Time	Time	Mean Difference	SE	df	t-statistic	*p*_bonferroni_
Time-Point 1	**Time-Point 2**	0.438	0.137	28	3.205	0.034
	**Time-Point 3**	0.625	0.137	28	4.578	< 0 0.001
	**Time-Point 4**	0.438	0.137	28	3.205	0.034
	Time-Point 5	0.063	0.137	28	0.458	1.000
Time-Point 2	Time-Point 3	0.188	0.137	28	1.373	1.000
	Time-Point 4	0.000	0.137	28	0.000	1.000
	Time-Point 5	-0.375	0.137	28	-2.747	0.104
Time-Point 3	Time-Point 4	-0.188	0.137	28	-1.373	1.000
	**Time-Point 5**	-0.563	0.137	28	-4.120	0.003
Time-Point 4	Time-Point 5	-0.375	0.137	28	-2.747	0.104

According to Fig. 3, there are notable changes in MAS levels between time-points 1, 2, and 3. However, MAS levels tend to increase over time and return to baseline by the fourth and fifth sessions. These results suggest that the trajectory of MAS scores over time is opposite to that of the TOI% measured with NIRS

### Correlation between NIRS and MAS

**Fig. 4:**
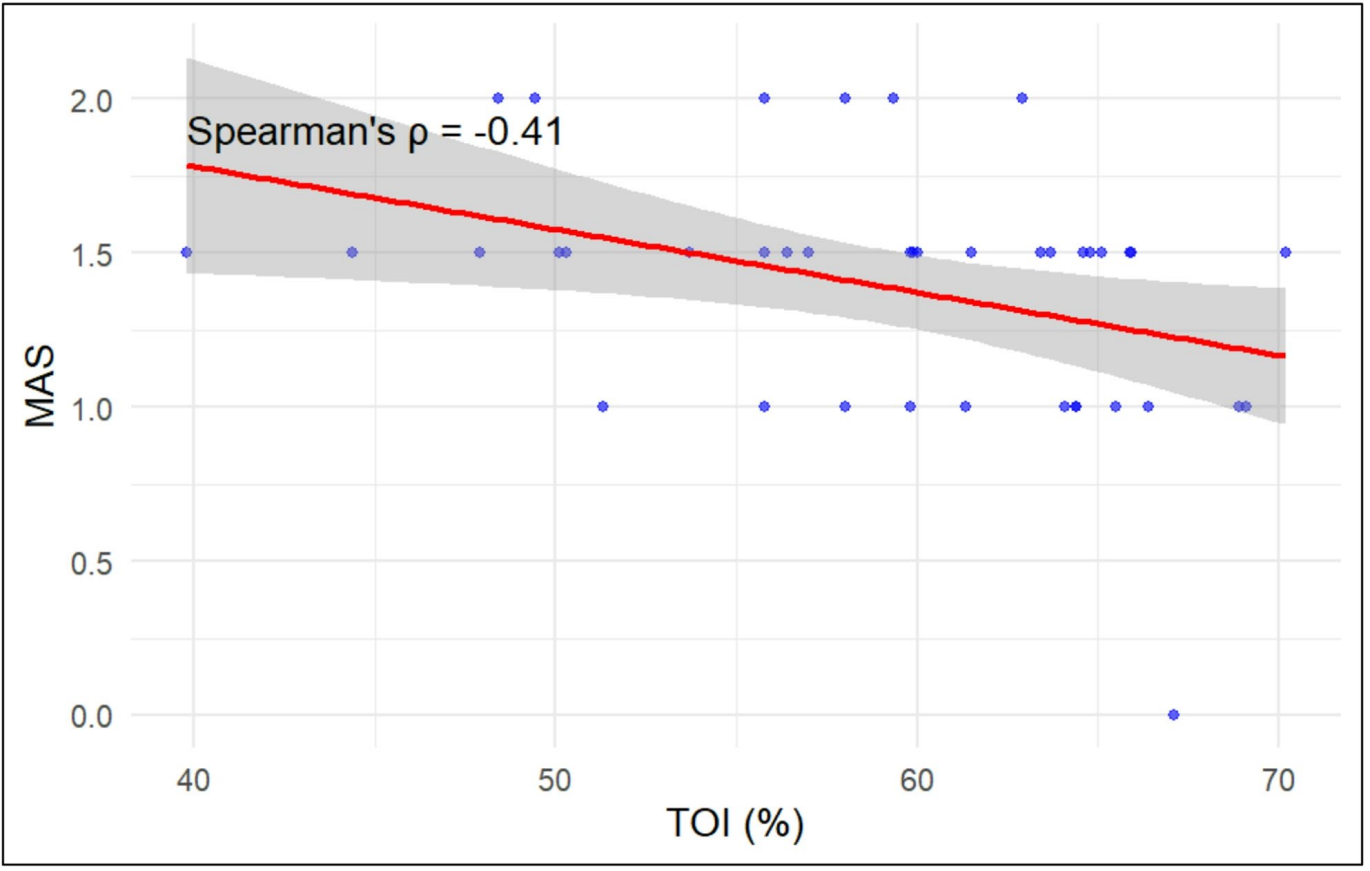
Scatterplot between NIRS-derived TOI% and MAS scores with linear regression line

Spearman’s rho correlation was used to measure the strength and direction of the relationship between TOI% (Muscle oxygen changes over time) and spasticity levels driven from MAS. The correlation coefficient of *r* = -0.409 indicates a moderate negative linear relationship between NIRS-derived oxygenation and the spasticity levels driven by MAS (*p* < 0.05) (Cohen, 1988). Figure 4 presents the scatter plot with the regression line between TOI% and MAS, graphically displaying the negative linear relationship between NIRS-derived TOI% and MAS scores. These results confirmed the reverse direction of MAS scores over time compared to the TOI% using NIRS, indicating that with increased muscle oxygen, spasticity levels driven by MAS decrease.

## Discussion

This pilot study investigated the feasibility of using NIRS as a non-invasive tool to assess and monitor muscle spasticity in children with CP undergoing BoNT-A treatment. Our findings demonstrate that NIRS-derived muscle oxygenation levels significantly increase following BoNT-A injection, peaking between 6 and 10 weeks and three months and then declining by six months. These changes paralleled reductions in spasticity as measured by the MAS and showed a significant negative correlation with MAS scores. While this supports the physiological relevance of NIRS-derived oxygenation measures, interpretation is constrained by the known limitations of MAS as a clinical comparator. Accordingly, these results should be regarded as preliminary evidence that NIRS has potential as an exploratory biomarker of treatment response, pending validation against more objective physiological measures.

### Principal findings

The study revealed a clear temporal trend in muscle tissue oxygenation, with TOI% values increasing after BoNT-A injection, reaching their highest levels at 6–10 weeks and three months post-treatment. This pattern is consistent with the known pharmacodynamics of BoNT-A, which typically exhibits maximal clinical efficacy during this timeframe before gradually waning. The subsequent decline in TOI% by six months corresponds with the expected reduction in therapeutic effects, reinforcing the physiological validity of the NIRS signal as a biomarker of treatment response [24, 25].

Importantly, MAS scores showed a similar trajectory, with significant reductions observed during the same post-injection window. The moderate negative correlation between TOI% and MAS scores supports the hypothesis that increased muscle perfusion and oxygenation are associated with reduced spastic hyperactivity. Despite the moderate strength of this correlation, the objective and continuous nature of NIRS-derived measurements may capture physiological changes that are not fully reflected in the MAS, owing to its ordinal scale and inherent subjectivity. Although the MAS is subject to inter-rater variability and limited sensitivity, it remains the most commonly used clinical tool for grading spasticity in both research and practice. Therefore, correlating TOI% with MAS offers an important initial reference point to assess the physiological relevance of NIRS measurements. However, future studies should incorporate complementary objective measures, such as surface EMG, biomechanical modelling, or elastography, to strengthen the validation of NIRS as a biomarker.

Our findings align with previous research indicating that spastic muscles exhibit altered perfusion and oxygen utilization patterns [27]. Sustained voluntary and involuntary contractions (spasms) elevate intramuscular pressure, which can mechanically compress capillaries and impair microvascular perfusion [28, 29] This vascular compromise reduces oxygen delivery and hinders metabolic exchange within the affected muscle tissue. Effective therapeutic interventions that reduce muscle overactivity can decrease intramuscular pressure, thereby restoring capillary patency and improving local blood flow and oxygen availability. Consequently, NIRS-derived measurements often demonstrate improved muscle oxygenation and hemodynamic responses following a reduction in spasticity. Conversely, increased muscle tone or sustained contraction can exacerbate intramuscular pressure, leading to further compromise of tissue perfusion and oxygen utilization.

Prior studies have also demonstrated that BoNT-A-induced muscle relaxation improves local blood flow and metabolic efficiency, which is reflected in increased tissue oxygenation [30]. These findings indicate that NIRS has potential as an exploratory, physiologically based biomarker for monitoring changes related to spasticity treatment. However, the current study does not establish threshold NIRS values for specific spasticity levels, nor does it validate NIRS as a standalone clinical tool. Larger studies are needed to determine clinically meaningful cut-off ranges and to verify the relationship between NIRS-derived parameters and established clinical scales.

### Clinical implications

This study contributes to a growing body of literature advocating for the integration of physiological monitoring tools in neurorehabilitation [11–14, 30]. NIRS offers several advantages in this context: it is non-invasive, portable, real-time, and relatively easy to administer in pediatric populations. By enabling continuous, quantitative tracking of muscle oxygenation, NIRS may inform the timing and dosage of repeat BoNT-A injections, improve individualized treatment planning, and provide objective outcome measures for research trials.

Furthermore, the observed decline in muscle oxygenation at six months suggests the need for re-evaluating spasticity and treatment needs at regular intervals to maintain therapeutic benefits. Incorporating NIRS into standard care protocols could help clinicians determine individualized re-injection schedules and tailor interventions based on real-time muscle physiology, enabling a more patient-specific approach to spasticity management that optimizes treatment precision and long-term functional outcomes.

### Limitations

While this study highlights the potential of NIRS as an objective tool for monitoring muscle spasticity, certain limitations must be acknowledged. The small sample size limits generalizability and precludes subgroup analyses by GMFCS level or muscle group. The use of MAS as the only comparator introduces subjectivity and restricts the precision of correlation analyses. Future studies should include parallel objective modalities for triangulation. Additionally, the absence of a non-spastic control group limits the interpretation of absolute TOI% values. No threshold values were derived to classify spasticity severity, a key step for clinical translation.

In addition, distinguishing spasticity from other forms of hypertonia, such as dystonia and rigidity, is crucial for accurate diagnosis and treatment. Spasticity is a velocity-dependent resistance to passive stretch, whereas dystonia involves involuntary muscle contractions leading to abnormal postures, and rigidity is characterized by non-velocity-dependent resistance. Recognizing these distinctions ensures precision in terminology and avoids conflating distinct motor disorders [31]. In this study, we use the term “hypertonia” to broadly describe increased muscle tone, emphasizing its role as an umbrella term that effectively differentiates dystonia from spasticity. While our study focused on spasticity as the clinical indication for BoNT-A, future studies should investigate how NIRS can distinguish between these conditions to refine clinical assessments.

### Future directions

Future research should involve larger, multi-center cohorts to confirm these preliminary results and explore variability across muscle groups, CP subtypes, and severity levels. Longitudinal studies assessing the predictive value of NIRS for functional outcomes, such as gait improvement, range of motion, or caregiver burden, would further enhance clinical relevance. Additionally, integrating NIRS with other modalities, such as EMG or ultrasound elastography, may provide a more comprehensive understanding of neuromuscular function and facilitate the development of multimodal biomarkers for spasticity and treatment monitoring in children with CP.

Finally, applying machine learning algorithms to analyze NIRS data may facilitate the development of automated, personalized treatment decision-support systems, enhancing their role in both clinical practice and research settings.

## Conclusion

This pilot study demonstrates that NIRS–based monitoring of muscle oxygenation is a feasible and non-invasive approach for assessing spasticity and tracking therapeutic response in children with CP undergoing BoNT-A treatment. The significant correlation between TOI% and MAS scores supports the physiological validity of NIRS-derived measures as potential objective indicators of spasticity severity. If validated in larger, controlled trials, NIRS could complement traditional clinical assessments by enabling real-time, quantitative evaluation of treatment effects, thereby enhancing precision in spasticity management and advancing personalized approaches in pediatric neurorehabilitation.

## Data Availability

The datasets used and/or analyzed during the current study are available from the corresponding author on reasonable request.
